# Supplementary material for: Potentiation of Antibiotic Activity of Aztreonam against Metallo-β-Lactamase-Producing Multidrug-Resistant Pseudomonas aeruginosa by 3-O-Substituted Difluoroquercetin Derivatives
Source: Pharmaceutics. 2024 Jan 28;16(2):185. doi: 10.3390/pharmaceutics16020185 (PMC10892423; doi:10.3390/pharmaceutics16020185)
Supplement: Supplementary file 1 [file pharmaceutics-16-00185-s001.zip › pharmaceutics-2820010-supplementary.pdf]

Supplementary Material

Potentiation of Antibiotic Activity of Aztreonam against Metallo-β-Lactamase-Producing Multidrug-Resistant *Pseudomonas aeruginosa* by 3-*O*-Substituted Difluoroquercetin Derivatives

Seongyeon Lee [1,2,†], Taegum Lee [1,2,†], Mi Kyoung Kim [2], Joong Hoon Ahn [2,3], Seri Jeong [4], Ki-Ho Park [5,*] and Youhoon Chong [2,3,*]

[1] Department of Bioscience and Biotechnology, Konkuk University, Hwayang-dong, Gwangjin-gu, Seoul 05029, Republic of Korea; tjddus98@konkuk.ac.kr (S.L.); co4121@konkuk.ac.kr (T.L.)
[2] Bio/Molecular Informatics Center, Konkuk University, Hwayang-dong, Gwangjin-gu, Seoul 05029, Republic of Korea; mkkim@konkuk.ac.kr (M.K.K.); jhanhn@konkuk.ac.kr (J.H.A.)
[3] Department of Integrative Bioscience and Biotechnology, Konkuk University, Hwayang-dong, Gwangjin-gu, Seoul 05029, Republic of Korea
[4] Department of Laboratory Medicine, Hallym University College of Medicine, Chuncheon 24252, Republic of Korea; hehebox73@hallym.or.kr
[5] Department of Infectious Disease, Kyung Hee University School of Medicine, Seoul 02447, Republic of Korea
* Correspondence: parkkiho@khu.ac.kr (K.-H.P.); chongy@konkuk.ac.kr (Y.C.); Tel.: +82-2-958-2966 (K.-H.P.); +82-2-2049-6100 (Y.C.)
† These authors contributed equally to this work.

Table of Contents

1. Synthesis and structural characterization of the title compounds..S3

2. **Figure S1.** Cell viability of human hepatoma HepG2 cells presented as percentage of control after 24 h exposure to different concentrations of **27**. The data represent the average values of three experiments (± SD)…………………………..…………....S20

3. **Table S1.** Synergistic anti-biofilm activity of ATM in combination with the efflux pump inhibitor (CCCP), β-lactamase inhibitor (AVI) or multitarget inhibitor **27** against 21 MBL-producing *P. aeruginosa* clinical isolates………………………..………S21

1. Synthesis and structural elucidation of the title compounds

Chemicals and reagents

Chemical reagents were purchased from Merck (4-*tert*-butylbenzyl bromide, 4-isopropylbenzyl bromide, and 2-chloro-*N*,*N*-dimethylethylamine hydrochloride), TCI [iodomethyl pivalate, chloromethyl isopropyl carbonate, diglycolamine, 1-(2-hydroxyethyl)ethyleneimine, 4-methylpiperazine-1-ethanol, 1-(2-chloroethyl)pyrrolidine hydrochloride, 2-(2-hydroxyethyl)-1-methylpyrrolidine, 1-bromo-3,3-dimethylbutane, and 1-bromo-3-methylbutane], Alfa Aesar [*tert*-butlacetyl chloride, 2-bromoethanol, diethylene glycol, triethylene glycol, 2-thiopheneethanol, *tert*-butyl bromoacetate, 1-piperazineethanol, 1-(2-chloroethyl)piperidine hydrochloride, 4-(2-hydroxyethyl)morpholine, 1,1,1-trifluoro-3-iodopropane, 2-cyclopropylethanol, 2-cyclopentylethanol, and 2-cyclohexylethanol] or Combi-Blocks [cyclopentanemethanol, 3-cyclopentyl-1-propanol, 2-(tetrahydro-2*H*-pyran-4-yl)ethanol, and 1-Boc-4-(2-hydroxyethyl)piperidine]. TLC was performed on silica gel-60 F254 purchased from Merck. Column chromatography was performed using silica gel-60 (220−440 mesh) for flash chromatography. Nuclear magnetic resonance spectra were recorded with a JNM-ECZ500R (Tokyo, Japan) at 500 MHz for ^{1}H NMR and 125 MHz for ^{13}C NMR using tetramethylsilane as an internal standard. Chemical shifts were reported as s (singlet), d (doublet), t (triplet), q (quartet), qu (quintet), sept (septet), m (multiplet), or br s (broad singlet). Coupling constants were reported in hertz (Hz). Chemical shifts were reported as parts per million (δ) relative to the solvent peak. Fast atom bombardment (FAB) mass spectra (FAB-MS) were obtained at the Korea Basic Science Institute (Daegu, Korea) and

reported in the form of *m*/*z* (intensity relative to base peak = 100).

General procedure for 3-O alkylation of di-F-Q

3’,4’-Difluoroquercetin (**1**) was prepared according to the previously reported procedure [48], and two different reaction conditions were applied to introduce various alkyl groups at 3-*O* position of **1**.

General procedure A. A solution of **1** (100 mg, 0.33 mmol, 1 eq.) in *N*,*N*-dimethylformamide (DMF, 3 ml) was treated with an appropriate alkyl halide (1.1 eq.) and K_2CO_3 (1.3 eq.). After stirring at 100 °C for 2 h, the reaction mixture was cooled to room temperature, treated with saturated aqueous NH_4Cl solution, and diluted with ethyl acetate (EtOAc). The organic layer was washed with brine, dried over $MgSO_4$, filtered, and concentrated under reduced pressure to give an oily residue, which was purified by column chromatography on silica gel to give the desired di-F-Q derivatives with various alkyl substituents at 3-*O* position. In some cases, the final products were obtained after removal of the protecting groups.

General procedure B. A cooled solution of **1** (100 mg, 0.33 mmol) in tetrahydrofuan (THF, 5 ml) was sequentially treated with an appropriate alcohol (1.5 eq.), PPh_3 (1.5 eq.), and diisopropyl azodicarboxylate (DIAD, 1.5 eq.). The resulting mixture was stirred for at room temperature for 1 h, and then concentrated under reduced pressure. The oily residue was purified by column chromatography on silica gel to give the desired 3-*O*-alkyl-di-F-Q.

1.1 2-(3,4-Difluorophenyl)-5,7-dihydroxy-4-oxo-4H-chromen-3-yl 3,3-dimethylbutanoate (**3**)**.** To a cooled solution of **1** (200 mg, 0.65 mmol) in DMF (5 ml) was added *tert*-butylacetyl chloride (0.12 ml, 0.85 mmol) and K_2CO_3 (135 mg, 9.8 mmol). After stirring for 1 h, the reaction mixture was treated with saturated aqueous NH_4Cl solution and diluted with EtOAc. The organic layer was

washed with brine, dried over $MgSO_4$, filtered, and concentrated under reduced pressure to give an oily residue, which was purified by column chromatography on silica gel (Hexanes:EtOAc = 6:1) to afford **3** (90 mg, 0.23 mmol, 34% yield) as yellow solid. ^{1}H NMR (500 MHz, $CDCl_3$) δ 12.08 (s, 1H), 7.73 (ddd, *J* = 2.2, 7.5, 11.0 Hz, 1H), 7.67-7.64 (m, 1H), 7.32 (ddd, *J* = 8.5, 8.5, 9.7 Hz, 1H), 6.27 (d, *J* = 2.1 Hz, 1H), 6.23 (d, *J* = 2.1 Hz, 1H), 2.53 (s, 2H), 1.12 (s, 9H); ^{13}C NMR (125 MHz, $CDCl_3$) δ 175.6, 170.6, 163.6, 162.1, 156.9, 154.6, 152.3 (dd, *J* = 12.0, 254.3 Hz), 150.4 (dd, *J* = 12.0, 249.6), 131.7, 126.4 (dd, *J* = 3.5, 6.0 Hz), 125.5 (dd, *J* = 3.5, 6.0 Hz), 118.2 (d, *J* = 19.3 Hz), 118.0 (d, *J* = 16.8 Hz), 105.4, 99.7, 94.5, 47.4, 31.1, 29.6; FAB-MS (*m/z*) found 405.4 $[M+H]^+$, calcd for $C_{21}H_{18}F_2O_6$ 404.1.

1.2 ((2-(3,4-Difluorophenyl)-5,7-dihydroxy-4-oxo-4H-chromen-3-yl)oxy)methyl pivalate (**4**). General procedure A was adopted using iodomethyl pivalate as an alkylating agent. After purification by column chromatography on silica gel (Hexanes:EtOAc = 6:1), the desired product **4** was obtained as yellow solid in 35% yield. ^{1}H NMR (500 MHz, acetone-*d*$_6$) δ 12.39 (s, 1H), 8.14 (ddd, *J* = 2.3, 7.9, 12.2 Hz, 1H), 8.05-8.02 (m, 1H), 7.55 (ddd, *J* = 8.6, 8.6, 10.4 Hz, 1H), 6.58 (d, *J* = 2.1 Hz, 1H), 6.32 (d, *J* = 2.1 Hz, 1H), 5.87 (s, 2H), 0.90 (s, 9H); ^{13}C NMR (125 MHz, acetone-*d*$_6$) δ 178.6, 177.6, 165.6, 163.1, 157.9, 154.1, 152.4 (dd, *J* = 13.1, 250.8 Hz), 150.7 (dd, *J* = 13.3, 244.9 Hz), 137.2, 128.5 (dd, *J* = 3.6, 7.1 Hz), 127.2 (dd, *J* = 3.6, 7.3 Hz), 119.3 (d, *J* = 19.1 Hz), 118.5 (d, *J* = 16.9 Hz), 105.8, 99.9, 94.9, 88.0, 39.1, 26.8; FAB-MS (*m/z*) found 421.4 $[M + H]^+$, calcd for $C_{21}H_{18}F_2O_7$ 420.1.

1.3 ((2-(3,4-Difluorophenyl)-5,7-dihydroxy-4-oxo-4H-chromen-3-yl)oxy)methyl isopropyl carbonate (**5**). General procedure A was adopted using chloromethyl isopropyl carbonate as an alkylating agent. After purification by column chromatography on silica gel (Hexanes:EtOAc = 6:1), the desired product **5** was obtained as yellow solid in 37% yield. ^{1}H NMR (500 MHz, acetone-*d*$_6$) δ 12.37 (s, 1H), 8.08 (ddd, *J* = 2.3, 7.9, 12.1 Hz, 1H), 7.99-7.96 (m, 1H), 7.55 (ddd, *J* =

8.6, 8.6, 10.4 Hz, 1H), 6.58 (d, J = 2.1 Hz, 1H), 6.32 (d, J = 2.1 Hz, 1H), 5.79 (s, 2H), 4.59 (sept, J = 6.3 Hz, 1H), 1.08 (d, J = 6.3 Hz, 6H); ^{13}C NMR (125 MHz, acetone-d_6) δ 178.4, 165.6, 163.1, 158.0, 154.8, 154.5, 152.4 (dd, J = 12.0, 250.8 Hz), 150.6 (dd, J = 12.0, 244.8 Hz), 136.8, 128.5-128.4 (m), 127.2 (dd, J = 3.6, 7.1 Hz), 119.2 (d, J = 19.3 Hz), 118.5 (d, J = 18.0 Hz), 105.9, 99.9, 94.9, 90.6, 73.1, 21.5; FAB-MS (*m/z*) found 423.3 $[M + H]^+$, calcd for $C_{20}H_{16}F_2O_8$ 422.1.

1.4 3-(4-Tert-butyl-benzyloxy)-2-(3,4-difluorophenyl)-5,7-dihydroxy-4H-chromen-4-one (**6**). General procedure A was adopted using 4-*tert*-butylbenzyl bromide as an alkylating agent. After purification by column chromatography on silica gel (Hexanes:acetone = 6:1), the desired product **6** was obtained as yellow solid in 67% yield. ^{1}H NMR (500 MHz, acetone-d_6) δ 12.66 (s, 1H), 7.90 (ddd, J = 2.2, 7.9, 12.1 Hz, 1H), 7.87-7.84 (m, 1H), 7.44 (ddd, J = 8.6, 8.6, 10.5 Hz, 1H), 7.32 (d, J = 8.3 Hz, 2H), 7.27 (d, J = 8.3 Hz, 2H), 6.53 (d, J = 2.0 Hz, 1H), 6.31 (d, J = 2.0 Hz, 1H), 5.13 (s, 2H), 1.28 (s, 9H); ^{13}C NMR (125 MHz, acetone-d_6) δ 179.6, 165.3, 163.3, 157.9, 154.8, 152.1, 152.1 (dd, J = 13.3, 250.8 Hz), 150.5 (dd, J = 13.3, 244.9 Hz), 138.6, 134.3, 129.8, 128.9 (dd, J = 3.6, 7.3 Hz), 126.9-126.8 (m), 125.9, 118.8 (d, J = 20.4 Hz), 118.2 (d, J = 18.0 Hz), 106.1, 99.7, 94.7, 74.9, 35.1, 31.5; FAB-MS (*m/z*) found 453.4 $[M + H]^+$, calcd for $C_{26}H_{22}F_2O_5$ 452.1.

1.5 2-(3,4-Difluorophenyl)-5,7-dihydroxy-3-(4-isopropyl-benzyloxy)-4H-chromen-4-one (**7**). General procedure A was adopted using 4-isopropylbenzyl bromide as an alkylating agent. After purification by column chromatography on silica gel (Hexanes:acetone = 6:1), the desired product **7** was obtained as pale yellow solid in 60% yield. ^{1}H NMR (500 MHz, acetone-d_6) δ 12.65 (s, 1H), 7.91 (ddd, J = 2.2, 7.9, 12.1 Hz, 1H), 7.87-7.84 (m, 1H), 7.43 (ddd, J = 8.6, 8.6, 10.5 Hz, 1H), 7.26 (d, J = 8.1 Hz, 2H), 7.15 (d, J = 8.1 Hz, 2H), 6.52 (d, J = 2.1 Hz, 1H), 6.30 (d, J = 2.1 Hz, 1H), 5.13 (s, 2H), 2.87 (sept, J = 7.0 Hz, 1H), 1.20 (d, J = 7.0 Hz, 6H); ^{13}C NMR (125 MHz, acetone-d_6) δ 179.6, 165.2, 163.2, 157.9, 154.7, 152.1 (dd, J = 13.1, 250.8 Hz), 150.5 (dd, J = 13.3, 244.8 Hz), 149.9, 138.6, 134.7, 130.1, 128.8 (dd, J = 3.6, 7.3 Hz), 127.0, 126.8 (dd, J = 3.6, 7.3 Hz), 118.7 (d,

J = 20.4), 118.2 (d, J = 18.0 Hz), 106.0, 99.6, 94.7, 74.9, 34.5, 24.2; FAB-MS (*m*/*z*) found 439.4 [M + H]$^+$, calcd for $C_{25}H_{20}F_2O_5$ 438.1.

1.6 2-(3,4-Difluorophenyl)-5,7-dihydroxy-3-(2-hydroxyethoxy)-4H-chromen-4-one (**8**). General procedure A was adopted using 2-bromoethanol as an alkylating agent. After purification by column chromatography on silica gel (Hexanes:acetone = 3:1), the desired product **8** was obtained as yellow solid in 43% yield. ^{1}H NMR (500 MHz, acetone-d_6) δ 12.49 (s, 1H), 8.29 (ddd, J = 2.2, 7.9, 12.4 Hz, 1H), 8.15-8.12 (m, 1H), 7.52 (ddd, J = 8.6, 8.6, 10.4 Hz, 1H), 6.57 (d, J = 2.1 Hz, 1H), 6.29 (d, J = 2.1 Hz, 1H), 4.23 (t, J = 4.6 Hz, 2H), 3.81 (t, J = 4.6 Hz, 2H); ^{13}C NMR (125 MHz, acetone-d_6) δ 179.8, 165.5, 163.1, 157.9, 154.0, 152.3 (dd, J = 13.3, 250.8 Hz), 150.8 (dd, J = 13.3, 244.9 Hz), 139.6, 128.9-128.8 (m), 126.8 (dd, J = 3.6, 7.3 Hz), 119.0 (d, J = 20.4 Hz), 118.5 (d, J = 16.8 Hz), 105.9, 99.7, 94.8, 75.5, 61.8; FAB-MS (*m*/*z*) found 351.3 [M + H]$^+$, calcd for $C_{17}H_{12}F_2O_6$ 350.1.

1.7 2-(3,4-Difluorophenyl)-5,7-dihydroxy-3-[2-(2-hydroxyethoxy)ethoxy]-4H-chromen-4-one (**9**). General procedure B was adopted using diethylene glycol as an alkylating agent. After purification by column chromatography on silica gel (Hexanes:acetone = 3:1), the desired product **9** was obtained as yellow solid in 35% yield. ^{1}H NMR (500 MHz, DMSO-d_6) δ 12.45 (s, 1H), 10.98 (s, 1H), 8.25 (ddd, J = 2.2, 8.0, 12.4 Hz, 1H), 8.08-8.05 (m, 1H), 7.63 (ddd, J = 8.7, 8.7, 10.5 Hz, 1H), 6.51 (d, J = 2.1 Hz, 1H), 6.22 (d, J = 2.1 Hz, 1H), 4.59 (t, J = 5.4 Hz, 1H), 4.26-4.25 (m, 2H), 3.63-3.61 (m, 2H), 3.44 (dt, J = 5.2, 5.3 Hz, 2H), 3.34 (t, J = 5.4 Hz, 2H); ^{13}C NMR (125 MHz, DMSO-d_6) δ 178.1, 164.6, 161.2, 156.4, 152.6, 150.8 (dd, J = 14.4, 253.1 Hz), 149.2 (dd, J = 13.3, 243.6 Hz), 137.9, 127.5 (dd, J = 3.6, 7.3 Hz), 126.2-126.1 (m), 118.0 (d, J = 21.6 Hz), 117.8 (d, J = 17.9 Hz), 104.4, 98.8, 94.0, 72.2, 71.6, 69.4, 60.1; FAB-MS (*m*/*z*) found 395.3 [M + H]$^+$, calcd for $C_{19}H_{16}F_2O_7$ 394.1.

1.8 2-(3,4-Difluorophenyl)-5,7-dihydroxy-3-{2-[2-(2-hydroxyethoxy)ethoxy]ethoxy}-4H-chromen-4-one (**10**). General procedure B was adopted using triethylene glycol as an alkylating agent. After purification by column chromatography on silica gel (Hexanes:acetone = 1:1), the desired product **5** was obtained as yellow solid in 38% yield. ^{1}H NMR (500 MHz, DMSO-d_6) δ 12.45 (s, 1H), 8.25 (ddd, *J* = 2.3, 8.0, 12.4 Hz, 1H), 8.06-8.03 (m, 1H), 7.63 (ddd, *J* = 8.7, 8.7, 10.5 Hz, 1H), 6.50 (d, *J* = 2.1 Hz, 1H), 6.22 (d, *J* = 2.1 Hz, 1H), 4.58 (s, 1H), 4.26-4.25 (m, 2H), 3.63-3.61 (m, 2H), 3.46-3.42 (m, 6H), 3.37 (t, *J* = 5.3 Hz, 2H); ^{13}C NMR (125 MHz, DMSO-d_6) δ 178.1, 164.6, 161.2, 156.4, 152.5, 150.7 (dd, *J* = 12.0, 250.8 Hz), 149.2 (dd, *J* = 13.1, 244.8 Hz), 137.9, 127.6-127.5 (m), 126.1 (dd, *J* = 3.6, 7.3 Hz), 118.0 (d, *J* = 20.4 Hz), 117.8 (d, *J* = 18.0 Hz), 104.4, 98.8, 94.0, 72.4, 71.5, 69.7, 69.7, 69.4, 60.2; FAB-MS (*m/z*) found 439.4 [M + H]$^+$, calcd for $C_{21}H_{20}F_2O_8$ 438.1.

1.9 3-[2-(2-Aminoethoxy)ethoxy]-2-(3,4-difluorophenyl)-5,7-dihydroxy-4H-chromen-4-one (**11**). The alkyl halide, benzyl (2-(2-iodoethoxy)ethyl)carbamate, was prepared as follows: To a cooled solution of diglycolamine (1 ml, 8.97 mmol) in THF (25 ml) were added triethylamine (TEA, 1.25 ml, 8.97 mmol) and benzyl chloroformate (1.4 ml, 9.87 mmol). The resulting mixture was stirred at room temperature for 1 h and then quenched with saturated aqueous NH_4Cl solution. The organic layer was washed with H_2O, dried over $MgSO_4$, filtered, and concentrated under reduced pressure to give a crude product, which was purified by column chromatography on silica gel (CH_2Cl_2:EtOAc = 1:1) to afford the Cbz-protected diglycolamine (1.53 g) as colorless oil. The Cbz-protected diglycolamine obtained above (1.53 g) was dissolved in CH_2Cl_2 (20 ml). To this solution, TEA (0.98 ml, 1.1 equiv) and methanesulfonyl chloride (MsCl, 0.55 ml) were added. After stirring for 30 min, the reaction mixture was quenched with saturated aqueous NH_4Cl solution. The organic layer was washed with H_2O, dried over $MgSO_4$, filtered, and concentrated under reduced pressure. The crude product dissolved in acetone (20 ml) was treated with NaI (1.11 g), and the resulting mixture was heated to reflux for 2.5 h. After cooling to room temperature, the reaction mixture was

diluted with EtOAc, washed with saturated aqueous $Na_2S_2O_3$ solution and brine, dried over $MgSO_4$, filtered, and concentrated under reduced pressure. The residue was purified by column chromatography on silica gel (Hexanes:EtOAc = 4:1) to afford benzyl (2-(2-iodoethoxy)ethyl)carbamate (1.18 g). General procedure A was adopted using the benzyl (2-(2-iodoethoxy)ethyl)carbamate obtained above, and the Cbz protecting group of the resulting di-F-Q derivative was removed as follows: The degassed suspension of the compound obtained above (43 mg, 0.09 mmol) and 10% Pd/C (4 mg) in a 1:1 mixture of CH_2Cl_2 (3 ml) and MeOH (3 ml) was vigorously stirred overnight under an atmosphere of H_2 (balloon). The reaction mixture was filtered through a short Celite pad and concentrated under reduced pressure. The crude product was purified by column chromatography in silica gel [CH_2Cl_2:MeOH:NH_4OH(aq.) = 90:10:1] to afford the desired product **11** (16 mg, 0.04 mmol, 47% yield) as yellow solid. ^1H NMR (500 MHz, methanol-d_4) δ 8.14 (ddd, J = 2.3, 7.9, 12.3 Hz, 1H), 7.97-7.94 (m, 1H), 7.44 (ddd, J = 8.6, 8.6, 10.3 Hz, 1H), 6.27 (d, J = 2.1 Hz, 1H), 6.09 (d, J = 2.1 Hz, 1H), 4.17-4.15 (m, 2H), 3.78-3.76 (m, 2H), 3.67-3.65 (m, 2H), 3.05-3.03 (m, 2H); ^{13}C NMR (125 MHz, methanol-d_4) δ 179.6, 168.5, 163.0, 158.6, 154.6, 153.0 (dd, J = 13.3, 250.8 Hz), 151.3 (dd, J = 13.1, 244.8 Hz), 139.5, 129.0 (dd, J = 3.6, 6.0 Hz), 126.8 (dd, J = 3.6, 7.1 Hz), 119.2 (d, J = 20.4 Hz), 118.8 (d, J = 18.0 Hz), 105.5, 100.8, 95.4, 73.1, 71.2, 68.3, 40.7; FAB-MS (m/z) found 394.3 $[M + H]^+$, calcd for $C_{19}H_{17}F_2NO_6$ 393.1.

1.10 2-(3,4-Difluorophenyl)-5,7-dihydroxy-3-[2-(thiophen-2-yl)ethoxy]-4H-chromen-4-one (**12**).

The alkylating agent, 2-(thiophen-2-yl)ethyl methanesulfonate, was prepared as follows: To a cooled solution of 2-(thiophen-2-yl)ethanol in CH_2Cl_2 were added TEA (1.1 eq.) and MsCl (1.1 eq.), and the resulting mixture was stirred for 1 h. The reaction mixture was quenched with saturated aqueous NH_4Cl solution, washed with H_2O, dried over $MgSO_4$, filtered, and concentrated under reduced pressure. The crude product was used for the next step without further purification. General procedure A was adopted using the compound obtained above as an alkylating agent. After

purification by column chromatography on silica gel (Hexanes:EtOAc = 6:1), the desired product **12** was obtained as yellow solid in 47% yield. ^{1}H NMR (500 MHz, acetone-d_6) δ 12.58 (s, 1H), 7.97 (ddd, J = 2.2, 7.9, 12.1 Hz, 1H), 7.90-7.86 (m, 1H), 7.39 (ddd, J = 8.6, 8.6, 10.5 Hz, 1H), 7.26 (dd, J = 1.5, 4.8 Hz, 1H), 6.94-6.91 (m, 2H), 6.53 (d, J = 2.1 Hz, 1H), 6.28 (d, J = 2.1 Hz, 1H), 4.40 (t, J = 6.6 Hz, 2H), 3.27 (t, J = 6.6 Hz, 2H); ^{13}C NMR (125 MHz, acetone-d_6) δ 179.5, 165.2, 163.2, 157.8, 154.0, 152.1 (dd, J = 13.3, 250.8 Hz), 150.6 (dd, J = 12.0, 244.8 Hz), 141.3, 139.3, 128.8-128.7 (m), 127.7, 126.8 (dd, J = 3.6, 7.3 Hz), 126.5, 124.7, 118.6 (d, J = 19.1 Hz), 118.4 (d, J = 18.0 Hz), 106.0, 99.7, 94.7, 73.8, 31.2; FAB-MS (m/z) found 417.3 $[M + H]^+$, calcd for $C_{21}H_{14}F_2O_5S$ 416.1.

1.11 2-(3,4-Difluorophenyl)-3-[2-(dimethylamino)ethoxy]-5,7-dihydroxy-4H-chromen-4-one (**13**). General procedure B was adopted using *N*,*N*-dimethylethanolamine as an alkylating agent. After trituration with acetone, the desired product **13** was obtained as yellow solid in 26% yield. ^{1}H NMR (500 MHz, DMSO-d_6) δ 12.29 (s, 1H), 8.11 (ddd, J = 2.1, 7.8, 11.7 Hz, 1H), 7.98-7.95 (m, 1H), 7.66 (ddd, J = 8.6, 8.6, 10.2 Hz, 1H), 6.57 (d, J = 2.0 Hz, 1H), 6.30 (d, J = 2.1 Hz, 1H), 4.28 (t, J = 4.7 Hz, 2H), 3.46 (t, J = 4.9 Hz, 2H), 2.89 (s, 6H); ^{13}C NMR (125 MHz, DMSO-d_6) δ 177.7, 165.0, 161.1, 156.6, 153.6, 151.0 (dd, J = 12.0, 250.8 Hz), 149.4 (dd, J = 13.1, 244.8 Hz), 137.3, 127.1 (dd, J = 3.6, 7.1 Hz), 126.3 (dd, J = 3.6, 7.1 Hz), 118.3 (d, J = 16.8 Hz), 117.9 (d, J = 19.1 Hz), 104.4, 99.2, 94.3, 66.4, 55.9, 42.6; FAB-MS (m/z) found 378.3 $[M + H]^+$, calcd for $C_{19}H_{17}F_2NO_5$ 377.1.

1.12 3-[2-(Aziridin-1-yl)ethoxy]-2-(3,4-difluorophenyl)-5,7-dihydroxy-4H-chromen-4-one (**14**). General procedure B was adopted using 1-(2-hydroxyethyl)ethyleneimine as an alkylating agent. After trituration with acetone, the desired product **14** was obtained as yellow solid in 28% yield. ^{1}H NMR (500 MHz, DMSO-d_6) δ 12.31 (s, 1H), 8.13 (ddd, J = 2.2, 7.9, 11.9 Hz, 1H), 7.99-7.96 (m, 1H), 7.65 (ddd, J = 8.7, 8.7, 10.4 Hz, 1H), 6.56 (d, J = 2.1 Hz, 1H), 6.29 (d, J = 2.1 Hz, 1H), 4.23

(t, J = 5.2 Hz, 2H), 3.93 (t, J = 6.2 Hz, 2H), 3.36 (t, J = 6.2 Hz, 2H), 3.28 (t, J = 5.2 Hz, 2H); ^{13}C NMR (125 MHz, DMSO-d_6) δ 177.8, 165.0, 161.1, 156.6, 153.4, 151.0 (dd, J = 12.0, 250.8 Hz), 149.4 (dd, J = 13.3, 244.8 Hz), 137.5, 127.2 (dd, J = 3.6, 7.1 Hz), 126.3-126.3 (m), 118.2 (d, J = 16.8 Hz), 117.9 (d, J = 19.1 Hz), 104.3, 99.1, 94.2, 68.3, 48.3, 46.8, 40.3; FAB-MS (*m*/*z*) found 376.3 $[M + H]^+$, calcd for $C_{19}H_{15}F_2NO_5$ 375.1.

1.13 2-((2-((2-(3,4-Difluorophenyl)-5,7-dihydroxy-4-oxo-4H-chromen-3-yl)oxy)ethyl)dimethylammonio)acetate (**15**)**.** To a solution of **2** (310 mg, 1.00 mmol) in DMF (10 ml) was added 2-chloro-*N*,*N*-dimethylethylamine hydrochloride (1.1 eq.) and K_2CO_3 (2.5 eq.) and stirred at 90 °C for 2 h. After cooling to room temperature, saturated aqueous NH_4Cl solution was added, and the reaction mixture was extracted with EtOAc. The combined organic layers were washed with brine, dried over $MgSO_4$, filtered, and concentrated under reduced pressure. The crude product was purified by column chromatography on silica gel (CH_2Cl_2:MeOH = 30:1 to 10:1) to afford the corresponding di-F-Q derivative as yellow solid in 65% yield. ^{1}H NMR (500 MHz, $CDCl_3$) δ 12.46 (s, 1H), 8.19 (ddd, J = 2.3, 7.8, 12.0 Hz, 1H), 7.98-7.95 (m, 1H), 7.31-7.26 (m, 1H), 6.64 (d, J = 2.2 Hz, 1H), 6.47 (d, J = 2.2 Hz, 1H), 5.25 (s, 2H), 4.18 (t, J = 5.5 Hz, 2H), 3.50 (s, 3H), 2.67 (t, J = 5.6 Hz, 2H), 2.30 (s, 6H).

The di-F-Q derivative obtained above (250 mg, 0.59 mmol) was dissolved in DMF (5 ml). The resulting solution was treated with *tert*-butyl bromoacetate (0.26 ml, 1.78 mmol) and stirred at room temperature for 12 h. The pale brown solid was filtered and washed with EtOAc to afford the corresponding ammonium bromide salt in 77% yield. ^{1}H NMR (500 MHz, DMSO-d_6) δ 12.18 (s, 1H), 8.14 (ddd, J = 2.2, 7.9, 11.9 Hz, 1H), 7.98-7.95 (m, 1H), 7.70 (ddd, J = 8.6, 8.6, 10.4 Hz, 1H), 6.94 (d, J = 2.1 Hz, 1H), 6.54 (d, J = 2.2 Hz, 1H), 5.35 (s, 2H), 4.59 (s, 2H), 4.43-4.41 (m, 2H), 4.04-4.02 (m, 2H), 3.41 (s, 3H), 3.36 (s, 6H), 1.38 (s, 9H).

The pale brown solid obtained above (100 mg, 0.16 mmol) was treated with 0.5 M HCl in MeOH (5 ml) and stirred at 70 °C overnight. After cooling to room temperature, the reaction mixture was

concentrated under reduced pressure, neutralized with saturated aqueous $NaHCO_3$, and purified by reverse-phase column chromatography on C18 (MeOH:H_2O = 3:1) to afford **15** as yellow solid in 30% yield. ^{1}H NMR (500 MHz, methanol-d_4) δ 7.93 (ddd, J = 2.2, 7.8, 11.8 Hz, 1H), 7.91-7.87 (m, 1H), 7.44 (ddd, J = 8.6, 8.6, 10.3 Hz, 1H), 6.08 (d, J = 2.0 Hz, 1H), 5.95 (d, J = 2.0 Hz, 1H), 4.33-4.32 (m, 2H), 4.24 (s, 2H), 4.12-4.11 (m, 2H), 3.49 (s, 6H); ^{13}C NMR (125 MHz, methanol-d_4) δ 178.9, 177.5, 169.3, 162.6, 159.3, 153.4, 152.7 (dd, J = 12.0, 250.8 Hz), 151.4 (dd, J = 13.3, 246.0 Hz), 138.0, 129.3 (dd, J = 3.5, 6.0 Hz), 126.7-126.6 (m), 118.9 (d, J = 18.0 Hz), 118.4 (d, J = 19.3 Hz), 104.2, 102.6, 97.8, 67.0, 66.0, 64.0, 53.1; FAB-MS (m/z) found 436.3 $[M + H]^+$, calcd for $C_{21}H_{19}F_2NO_7$ 435.1.

1.14 2-(3,4-Difluorophenyl)-5,7-dihydroxy-3-[2-(4-methylpiperazin-1-yl)ethoxy]-4H-chromen-4-one (**16**)**.** General procedure B was adopted using 4-methylpiperazine-1-ethanol as an alkylating agent. After trituration with EtOAc, the desired product **16** was obtained as off-white solid in 54% yield. ^{1}H NMR (500 MHz, D_2O) δ 7.66-7.60 (m, 2H), 7.29 (ddd, J = 8.6, 8.6, 10.0 Hz, 1H), 6.03 (d, J = 2.1 Hz, 1H), 6.00 (d, J = 2.1 Hz, 1H), 4.13 (t, J = 4.6 Hz, 2H), 3.79 (br s, 8H), 3.64 (t, J = 4.5 Hz, 2H), 3.10 (s, 3H); ^{13}C NMR (125 MHz, D_2O) δ 177.7, 163.5, 159.8, 156.1, 154.1, 151.8 (dd, J = 12.0, 253.1 Hz), 149.8 (dd, J = 13.1, 244.8 Hz), 136.8, 125.8 (dd, J = 3.6, 7.3 Hz), 125.7-125.6 (m), 118.1 (d, J = 18.0 Hz), 117.2 (d, J = 20.5 Hz), 104.3, 99.1, 94.5, 66.2, 56.7, 50.5, 49.5, 42.9; FAB-MS (m/z) found 433.4 $[M + H]^+$, calcd for $C_{22}H_{22}F_2N_2O_5$ 432.1.

1.15 2-(3,4-Difluorophenyl)-5,7-dihydroxy-3-[2-(piperazin-1-yl)ethoxy]-4H-chromen-4-one (**17**)**.** General procedure B was adopted using 1-piperazineethanol as an alkylating agent. After trituration with EtOAc, the desired product **17** was obtained as yellow solid in 46% yield. ^{1}H NMR (500 MHz, D_2O) δ 7.77 (ddd, J = 2.0, 7.7, 11.5 Hz, 1H), 7.73-7.71 (m, 1H), 7.37-7.32 (m, 1H), 6.23 (d, J = 2.0 Hz, 1H), 6.12 (d, J = 2.0 Hz, 1H), 4.14 (t, J = 4.8 Hz, 2H), 3.80-3.74 (m, 4H), 3.73-3.69 (m, 4H), 3.61 (t, J = 4.6 Hz, 2H); ^{13}C NMR (125 MHz, DMSO-d_6) δ 177.7, 165.0, 161.1, 156.6, 153.5,

151.0 (dd, J = 13.3, 250.8 Hz), 149.5 (dd, J = 13.3, 244.8 Hz), 137.4, 127.1-127.1 (m), 126.4-126.4 (m), 118.3 (d, J = 16.8 Hz), 117.9 (d, J = 19.1 Hz), 104.3, 99.2, 94.3, 66.3, 55.3, 48.3, 48.3; FAB-MS (*m*/*z*) found 419.4 $[M + H]^+$, calcd for $C_{21}H_{20}F_2N_2O_5$ 418.1.

1.16 2-(3,4-Difluorophenyl)-5,7-dihydroxy-3-[2-(pyrrolidin-1-yl)ethoxy]-4H-chromen-4-one (**18**). General procedure A was adopted using 1-(2-chloroethyl)pyrrolidine hydrochloride as an alkylating agent. After trituration with EtOH, the desired product **18** was obtained as pale yellow solid in 32% yield. ^{1}H NMR (500 MHz, DMSO-d_6) δ 12.32 (s, 1H), 11.18 (s, 1H), 10.31 (s, 1H), 8.13 (ddd, J = 2.2, 7.9, 11.8 Hz, 1H), 7.98-7.96 (m, 1H), 7.67 (ddd, J = 8.7, 8.7, 10.4 Hz, 1H), 6.56 (d, J = 2.1 Hz, 1H), 6.29 (d, J = 2.1 Hz, 1H), 4.27 (t, J = 4.8 Hz, 2H), 3.67 (br s, 2H), 3.56-3.50 (m, 2H), 3.11 (br s, 2H), 2.01 (br s, 2H), 1.93 (br s, 2H); ^{13}C NMR (125 MHz, DMSO-d_6) δ 177.7, 164.9, 161.1, 156.6, 153.6, 151.0 (dd, J = 12.0, 249.5 Hz), 149.4 (dd, J = 12.0, 244.8 Hz), 137.3, 127.7-127.7 (m), 126.3-126.3 (m), 118.3 (d, J = 18.0 Hz), 117.9 (d, J = 19.3 Hz), 104.4, 99.1, 94.3, 67.4, 53.5, 53.3, 22.6; FAB-MS (*m*/*z*) found 404.4 $[M + H]^+$, calcd for $C_{21}H_{19}F_2NO_5$ 403.1.

1.17 2-(3,4-Difluorophenyl)-5,7-dihydroxy-3-[2-(piperidin-1-yl)ethoxy]-4H-chromen-4-one (**19**). General procedure A was adopted using 1-(2-chloroethyl)piperidine hydrochloride as an alkylating agent. After trituration with EtOH, the desired product **19** was obtained as yellow solid in 34% yield. ^{1}H NMR (500 MHz, DMSO-d_6) δ 12.29 (s, 1H), 11.25 (s, 1H), 10.25 (s, 1H), 8.11 (ddd, J = 2.2, 7.8, 11.8 Hz, 1H), 7.97-7.94 (m, 1H), 7.67 (ddd, J = 8.6, 8.6, 10.5 Hz, 1H), 6.57 (d, J = 2.0 Hz, 1H), 6.31 (d, J = 2.1 Hz, 1H), 4.33 (t, J = 4.7 Hz, 2H), 3.57-3.51 (m, 2H), 3.45-3.41 (m, 2H), 3.02-2.95 (m, 2H), 1.83-1.75 (m, 4H), 1.73-1.69 (m, 1H), 1.42-1.32 (m, 1H); ^{13}C NMR (125 MHz, DMSO-d_6) δ 178.1, 165.0, 161.2, 156.4, 152.3, 150.0 (dd, J = 12.0, 250.8 Hz), 149.2 (dd, J = 13.3, 244.8 Hz), 138.0, 127.7-127.7 (m), 126.0-126.0 (m), 118.1 (d, J = 19.3 Hz), 117.9 (d, J = 16.9 Hz), 104.3, 99.0, 94.0, 68.6, 57.7, 53.8, 34.0, 24.9; FAB-MS (*m*/*z*) found 418.4 $[M + H]^+$, calcd for $C_{22}H_{21}F_2NO_5$ 417.1.

1.18 2-(3,4-Difluorophenyl)-5,7-dihydroxy-3-(2-morpholinoethoxy)-4H-chromen-4-one (**20**). General procedure B was adopted using 4-(2-hydroxyethyl)morpholine as an alkylating agent. After trituration with EtOAc, the desired product **20** was obtained as off-white solid in 60% yield. ^{1}H NMR (500 MHz, DMSO-d_6) δ 12.26 (s, 1H), 11.25 (s, 1H), 11.15 (s, 1H), 8.11 (ddd, *J* = 2.2, 7.8, 11.8 Hz, 1H), 7.98-7.95 (m, 1H), 7.65 (ddd, *J* = 8.6, 8.6, 10.4 Hz, 1H), 6.57 (d, *J* = 2.1 Hz, 1H), 6.30 (d, *J* = 2.1 Hz, 1H), 4.35 (t, *J* = 4.6 Hz, 2H), 4.00-3.98 (m, 2H), 3.87-3.83 (m, 2H), 3.58-3.52 (m, 4H), 3.24-3.19 (m, 2H); ^{13}C NMR (125 MHz, DMSO-d_6) δ 177.7, 165.0, 161.1, 156.5, 153.6, 151.0 (dd, *J* = 13.1, 250.8 Hz), 149.4 (dd, *J* = 13.3, 246.0 Hz), 137.3, 127.1-127.1 (m), 126.4-126.3 (m), 118.2 (d, *J* = 16.8 Hz), 117.9 (d, *J* = 19.1 Hz), 104.3, 99.1, 94.3, 66.0, 63.2, 55.4, 51.3; FAB-MS (*m*/*z*) found 420.3 $[M + H]^+$, calcd for $C_{21}H_{19}F_2NO_6$ 419.1.

1.19 2-(3,4-Difluorophenyl)-5,7-dihydroxy-3-[2-(1-methylpyrrolidin-2-yl)ethoxy]-4H-chromen-4-one (**21**). General procedure B was adopted using 2-(2-hydroxyethyl)-1-methylpyrrolidine as an alkylating agent. After trituration with EtOAc, the desired product **21** was obtained as yellow solid in 35% yield. ^{1}H NMR (500 MHz, methanol-d_4) δ 8.04 (ddd, *J* = 2.2, 7.7, 11.8 Hz, 1H), 7.99-7.95 (m, 1H), 7.51 (ddd, *J* = 8.6, 8.6, 10.2 Hz, 1H), 6.48 (d, *J* = 2.1 Hz, 1H), 6.26 (d, *J* = 2.2 Hz, 1H), 4.07 (t, *J* = 5.2 Hz, 2H), 3.87 (ddd, *J* = 5.5, 7.7, 11.7 Hz, 1H), 3.74-3.68 (m, 1H), 3.28-3.22 (m, 1H), 3.06 (s, 3H), 2.45-2.38 (m, 1H), 2.26 (td, *J* = 5.2, 5.5 Hz, 2H), 2.23-2.16 (m, 2H), 2.03-1.95 (m, 1H); ^{13}C NMR (125 MHz, methanol-d_4) δ 180.1, 167.0, 163.0, 158.7, 155.8, 153.3 (dd, *J* = 13.1, 252.0 Hz), 151.6 (dd, *J* = 12.0, 242.4 Hz), 139.4, 128.6-128.6 (m), 127.1-127.0 (m), 119.2 (d, *J* = 18.0 Hz), 118.9 (d, *J* = 20.4 Hz), 105.8, 100.4, 95.3, 70.6, 69.0, 57.5, 40.2, 30.4, 29.7, 22.3; FAB-MS (*m*/*z*) found 418.4 $[M + H]^+$, calcd for $C_{22}H_{21}F_2NO_5$ 417.1.

1.20 2-(3,4-Difluorophenyl)-5,7-dihydroxy-3-(3,3,3-trifluoropropoxy)-4H-chromen-4-one (**22**). General procedure A was adopted using 1,1,1-trifluoro-3-iodopropane as an alkylating agent. After

purification by column chromatography on silica gel (Hexanes:EtOAc = 4:1), the desired product **22** was obtained as pale yellow solid in 50% yield. ^{1}H NMR (500 MHz, acetone-d_6) δ 12.48 (s, 1H), 8.07 (ddd, *J* = 2.2, 7.9, 12.1 Hz, 1H), 8.01-7.98 (m, 1H), 7.54 (ddd, *J* = 8.6, 8.6, 10.4 Hz, 1H), 6.54 (d, *J* = 2.1 Hz, 1H), 6.29 (d, *J* = 2.1 Hz, 1H), 4.39 (t, *J* = 6.3 Hz, 2H), 2.78 (qt, *J* = 4.9, 6.3 Hz, 2H); ^{13}C NMR (125 MHz, acetone-d_6) δ 179.3, 165.4, 161.2, 157.9, 154.3, 152.3 (dd, *J* = 13.3, 250.9 Hz), 150.7 (dd, *J* = 13.1, 245.9 Hz), 139.0, 128.7-128.6 (m), 127.5 (q, *J* = 273.6 Hz), 126.9 (dd, *J* = 3.6, 7.3 Hz), 118.8 (d, *J* = 19.1 Hz), 118.6 (d, *J* = 18.0 Hz), 106.0, 99.8, 94.8, 66.2 (q, *J* = 3.8 Hz), 35.0 (q, *J* = 22.8 Hz); FAB-MS (*m*/*z*) found 403.3 [M + H]$^+$, calcd for $C_{18}H_{11}F_5O_5$ 402.1.

1.21 2-(3,4-Difluorophenyl)-3-(3,3-dimethylbutoxy)-5,7-dihydroxy-4H-chromen-4-one (**23**). General procedure A was adopted using 1-bromo-3,3-dimethylbutane as an alkylating agent. After purification by column chromatography on silica gel (Hexanes:EtOAc = 6:1), the desired product **23** was obtained as yellow solid in 67% yield. ^{1}H NMR (500 MHz, acetone-d_6) δ 12.62 (s, 1H), 8.11 (ddd, *J* = 2.3, 7.9, 12.3 Hz, 1H), 8.05-8.02 (m, 1H), 7.55 (ddd, *J* = 8.6, 8.6, 10.4 Hz, 1H), 4.20 (t, *J* = 7.5 Hz, 2H), 1.72 (t, *J* = 7.5 Hz, 2H), 0.95 (s, 9H); ^{13}C NMR (125 MHz, acetone-d_6) δ 179.7, 165.2, 163.2, 157.8, 153.9, 152.2 (dd, *J* = 13.3, 250.8 Hz), 150.7 (dd, *J* = 13.3, 244.9 Hz), 139.8, 129.0 (dd, *J* = 3.6, 7.3 Hz), 126.8-126.7 (m), 118.6 (d, *J* = 20.4 Hz), 118.6 (d, *J* = 16.8 Hz), 106.1, 99.6, 94.7, 71.2, 44.0, 30.1, 29.9; FAB-MS (*m*/*z*) found 391.4 [M + H]$^+$, calcd for $C_{21}H_{20}F_2O_5$ 390.1.

1.22 2-(3,4-difluorophenyl)-5,7-dihydroxy-3-(3-methylbutoxy)-4H-chromen-4-one (**24**). General procedure A was adopted using 1-bromo-3-methylbutane as an alkylating agent. After purification by column chromatography on silica gel (Hexanes:EtOAc = 6:1), the desired product **24** was obtained as yellow solid in 70% yield. ^{1}H NMR (500 MHz, acetone-d_6) δ 12.63 (s, 1H), 8.11 (ddd, *J* = 2.2, 7.9, 12.2 Hz, 1H), 8.04-8.01 (m, 1H), 7.56 (ddd, *J* = 8.6, 8.6, 10.5 Hz, 1H), 6.54 (d, *J* = 2.1 Hz, 1H), 6.29 (d, *J* = 2.0 Hz, 1H), 4.15 (t, *J* = 6.6 Hz, 2H), 1.79 (t sept, *J* = 6.6, 6.7 Hz, 1H), 1.61

(dt, J = 6.7, 6.8 Hz, 2H), 0.91 (d, J = 6.7 Hz, 6H); ^{13}C NMR (125 MHz, acetone-d_6) δ 179.6, 165.2, 163.2, 157.8, 154.0, 152.2 (dd, J = 13.1, 250.8 Hz), 150.7 (dd, J = 13.3, 244.8 Hz), 139.7, 129.0-128.9 (m), 126.7-126.7 (m), 118.6 (d, J = 19.1 Hz), 118.5 (d, J = 15.6 Hz), 106.1, 99.6, 94.7, 71.9, 39.6, 25.5, 22.7; FAB-MS (*m*/*z*) found 377.4 [M + H]$^+$, calcd for $C_{20}H_{18}F_2O_5$ 376.1.

1.23 3-(2-Cyclopropylethoxy)-2-(3,4-difluorophenyl)-5,7-dihydroxy-4H-chromen-4-one (**25**). The alkylating agent, 2-cyclopropylethyl methanesulfonate, was prepared as follows: To a cooled solution of 2-cyclopropylethanol in CH_2Cl_2 were added TEA (1.1 eq.) and MsCl (1.1 eq.), and the resulting mixture was stirred for 1 h. The reaction mixture was quenched with saturated aqueous NH_4Cl solution, washed with H_2O, dried over $MgSO_4$, filtered, and concentrated under reduced pressure. The crude product was used for the next step without further purification. General procedure A was adopted using the compound obtained above as an alkylating agent. After purification by column chromatography on silica gel (Hexanes:acetone = 6:1), the desired product **25** was obtained as yellow solid in 51% yield. ^{1}H NMR (500 MHz, acetone-d_6) δ 12.61 (s, 1H), 8.13 (ddd, J = 2.2, 7.9, 12.2 Hz, 1H), 8.06-8.03 (m, 1H), 7.54 (ddd, J = 8.6, 8.6, 10.4 Hz, 1H), 6.53 (d, J = 2.1 Hz, 1H), 6.27 (d, J = 2.1 Hz, 1H), 4.18 (t, J = 6.7 Hz, 2H), 1.63 (td, J = 6.7, 6.8 Hz, 2H), 0.85-0.77 (m, 1H), 0.46-0.42 (m, 2H), 0.10-0.07 (m, 2H); ^{13}C NMR (125 MHz, acetone-d_6) δ 179.6, 165.2, 163.2, 157.8, 153.9, 152.2 (dd, J = 12.0, 250.8 Hz), 150.7 (dd, J = 13.1, 244.8 Hz), 139.7, 129.0-128.9 (m), 126.7 (dd, J = 3.6, 7.3 Hz), 118.7 (d, J = 20.5 Hz), 118.5 (d, J = 16.8 Hz), 106.1, 99.6, 94.7, 73.5, 35.6, 8.4, 4.7; FAB-MS (*m*/*z*) found 375.3 [M + H]$^+$, calcd for $C_{20}H_{16}F_2O_5$ 374.1.

1.24 3-(2-Cyclopentylethoxy)-2-(3,4-difluorophenyl)-5,7-dihydroxy-4H-chromen-4-one (**26**). General procedure B was adopted using 2-cyclopentylethanol as an alkylating agent. After purification by column chromatography on silica gel (Hexanes:EtOAc = 6:1), the desired product **26** was obtained as yellow solid in 70% yield. ^{1}H NMR (500 MHz, $CDCl_3$) δ 12.52 (s, 1H), 7.97

(ddd, *J* = 2.2, 7.7, 11.7 Hz, 1H), 7.90-7.86 (m, 1H), 7.29 (ddd, *J* = 8.6, 8.6, 9.6 Hz, 1H), 6.45 (d, *J* = 2.2 Hz, 1H), 6.34 (d, *J* = 2.1 Hz, 1H), 4.00 (t, *J* = 7.0 Hz, 2H), 1.84 (t qu, *J* = 7.5, 8.3 Hz, 1H), 1.73 (td, *J* = 7.0, 7.1 Hz, 2H), 1.73-1.67 (m, 2H), 1.61-1.53 (m, 2H), 1.52-1.43 (m, 2H), 1.08-1.01 (m, 2H); ^{13}C NMR (125 MHz, $CDCl_3$) δ 179.1, 163.2, 162.2, 157.0, 154.0, 151.9 (dd, *J* = 13.1, 254.4 Hz), 150.2 (dd, *J* = 13.3, 248.4 Hz), 139.1, 127.5-127.4 (m), 125.5 (dd, *J* = 3.6, 6.0 Hz), 118.1 (d, *J* = 20.5 Hz), 117.7 (d, *J* = 16.9 Hz), 105.9, 99.6, 94.3, 73.2, 36.6, 36.2, 32.7, 25.1; FAB-MS (*m*/*z*) found 403.4 $[M + H]^+$, calcd for $C_{22}H_{20}F_2O_5$ 402.1.

1.25 3-(2-Cyclohexylethoxy)-2-(3,4-difluorophenyl)-5,7-dihydroxy-4H-chromen-4-one (**27**). General procedure B was adopted using 2-cyclohexylethanol as an alkylating agent. After purification by column chromatography on silica gel (Hexanes:EtOAc = 10:1), the desired product **27** was obtained as yellow solid in 66% yield. ^{1}H NMR (500 MHz, $CDCl_3$) δ 12.56 (s, 1H), 7.97 (ddd, *J* = 2.2, 7.7, 11.6 Hz, 1H), 7.89-7.85 (m, 1H), 7.30 (ddd, *J* = 8.6, 8.6, 9.7 Hz, 1H), 6.42 (d, *J* = 2.1 Hz, 1H), 6.31 (d, *J* = 2.1 Hz, 1H), 4.06 (t, *J* = 6.8 Hz, 2H), 1.68-1.61 (m, 5H), 1.59 (dt, *J* = 6.8, 6.8 Hz, 2H), 1.42-1.34 (m, 1H), 1.21-1.07 (m, 3H), 0.90-0.83 (m, 2H); ^{13}C NMR (125 MHz, $CDCl_3$) δ 179.1, 162.8, 162.4, 156.9, 153.8, 151.8 (dd, *J* = 13.1, 254.4 Hz), 150.2 (dd, *J* = 13.3, 247.3 Hz), 139.1, 127.6-127.5 (m), 125.5 (dd, *J* = 3.6, 6.0 Hz), 118.2 (d, *J* = 19.1 Hz), 117.6 (d, *J* = 18.0 Hz), 106.1, 99.5, 94.2, 71.6, 37.5, 34.4, 33.3, 26.6, 26.3; FAB-MS (*m*/*z*) found 417.4 $[M + H]^+$, calcd for $C_{23}H_{22}F_2O_5$ 416.1.

1.26 3-(Cyclopentylmethoxy)-2-(3,4-difluorophenyl)-5,7-dihydroxy-4H-chromen-4-one (**28**). General procedure B was adopted using cyclopentanemethanol as an alkylating agent. After purification by column chromatography on silica gel (Hexanes:acetone = 6:1), the desired product **28** was obtained as yellow solid in 34% yield. ^{1}H NMR (500 MHz, acetone-d_6) δ 12.61 (s, 1H), 8.09 (ddd, *J* = 2.2, 7.9, 12.2 Hz, 1H), 8.02-7.99 (m, 1H), 7.54 (ddd, *J* = 8.6, 8.6, 10.5 Hz, 1H), 6.52 (d, *J* = 2.1 Hz, 1H), 6.27 (d, *J* = 2.1 Hz, 1H), 3.98 (d, *J* = 7.0 Hz, 2H), 2.30 (t qu, *J* = 7.5, 7.6 Hz,

1H), 1.77-1.70 (m, 2H), 1.61-1.51 (m, 4H), 1.36-1.29 (m, 2H); ^{13}C NMR (125 MHz, acetone-d_6) δ 179.6, 165.2, 163.2, 157.8, 153.9, 152.2 (dd, *J* = 12.0, 250.8 Hz), 150.7 (dd, *J* = 12.0, 244.8 Hz), 139.6, 129.0-128.9 (m), 126.8 (dd, *J* = 3.6, 7.3 Hz), 118.8 (d, *J* = 20.4 Hz), 118.5 (d, *J* = 18.0 Hz), 106.1, 99.6, 94.7, 77.5, 40.6, 30.0, 26.1; FAB-MS (*m*/*z*) found 389.3 [M + H]$^+$, calcd for $C_{21}H_{18}F_2O_5$ 388.1.

1.27 3-(3-Cyclopentylpropoxy)-2-(3,4-difluorophenyl)-5,7-dihydroxy-4H-chromen-4-one (**29**). General procedure B was adopted using 3-cyclopentyl-1-propanol as an alkylating agent. After purification by column chromatography on silica gel (Hexanes:EtOAc = 4:1), the desired product **29** was obtained as yellow solid in 55% yield. ^{1}H NMR (500 MHz, acetone-d_6) δ 12.61 (s, 1H), 8.10 (ddd, *J* = 2.2, 7.9, 12.2 Hz, 1H), 8.02-7.99 (m, 1H), 7.54 (ddd, *J* = 8.6, 8.6, 10.4 Hz, 1H), 6.52 (d, *J* = 2.1 Hz, 1H), 6.27 (d, *J* = 2.1 Hz, 1H), 4.12 (t, *J* = 6.5 Hz, 2H), 1.78-1.68 (m, 5H), 1.62-1.55 (m, 2H), 1.53-1.45 (m, 2H), 1.40-1.36 (m, 2H), 1.09-1.02 (m, 2H); ^{13}C NMR (125 MHz, acetone-d_6) δ 179.6, 165.2, 163.2, 157.8, 154.0, 152.2 (dd, *J* = 12.0, 250.8 Hz), 150.7 (dd, *J* = 13.3, 244.9 Hz), 139.6, 129.0 (dd, *J* = 3.5, 6.0 Hz), 126.8 (dd, *J* = 3.6, 6.0 Hz), 118.8 (d, *J* = 20.4 Hz), 118.5 (d, *J* = 16.9 Hz), 106.1, 99.6, 94.7, 73.8, 40.6, 33.2, 33.1, 29.9, 25.7; FAB-MS (*m*/*z*) found 417.4 [M + H]$^+$, calcd for $C_{23}H_{22}F_2O_5$ 416.1.

1.28 2-(3,4-Difluorophenyl)-5,7-dihydroxy-3-[2-(tetrahydro-2H-pyran-4-yl)ethoxy]-4H-chromen-4-one (**30**). General procedure B was adopted using 2-(tetrahydro-2*H*-pyran-4-yl)ethanol as an alkylating agent. After purification by column chromatography on silica gel (Hexanes:acetone = 3:1), the desired product **30** was obtained as pale yellow solid in 56% yield. ^{1}H NMR (500 MHz, DMSO-d_6) δ 12.46 (s, 1H), 8.04 (ddd, *J* = 2.2, 7.9, 12.0 Hz, 1H), 7.89-7.86 (m, 1H), 7.66 (ddd, *J* = 8.6, 8.6, 10.6 Hz, 1H), 6.45 (d, *J* = 2.1 Hz, 1H), 6.21 (d, *J* = 2.1 Hz, 1H), 4.03 (t, *J* = 6.0 Hz, 2H), 3.80-3.77 (m, 2H), 3.16 (dt, *J* = 2.0, 11.9 Hz, 2H), 1.61-1.50 (m, 1H), 1.53 (t, *J* = 5.9 Hz, 2H), 1.49-1.46 (m, 2H), 1.13-1.05 (m, 2H); ^{13}C NMR (125 MHz, DMSO-d_6) δ 178.1, 164.6, 161.3,

156.5, 153.1, 150.7 (dd, J = 12.0, 249.5 Hz), 149.2 (dd, J = 13.1, 244.8 Hz), 138.0, 127.6 (dd, J = 3.6, 7.3 Hz), 126.1-126.0 (m), 118.0 (d, J = 18.0 Hz), 117.8 (d, J = 19.3 Hz), 104.5, 98.8, 94.0, 69.6, 67.0, 36.3, 32.4, 31.1; FAB-MS (*m*/*z*) found 419.3 $[M + H]^+$, calcd for $C_{22}H_{20}F_2O_6$ 418.1.

1.29 2-(3,4-Difluorophenyl)-5,7-dihydroxy-3-[2-(piperidin-4-yl)ethoxy]-4H-chromen-4-one (**31**). General procedure B was adopted using 1-Boc-4-(2-hydroxyethyl)piperidine as an alkylating agent. After trituration with Et_2O, the desired product **31** was obtained as yellow solid in 51% yield. ^{1}H NMR (500 MHz, DMSO-d_6) δ 12.46 (s, 1H), 11.13 (s, 1H), 8.90-8.88 (m, 1H), 8.68-8.62 (m, 1H), 8.07 (ddd, J = 2.2, 7.9, 12.0 Hz, 1H), 7.92-7.89 (m, 1H), 7.68 (ddd, J = 8.6, 8.6, 10.5 Hz, 1H), 6.52 (d, J = 2.1 Hz, 1H), 6.26 (d, J = 2.1 Hz, 1H), 4.03 (t, J = 6.2 Hz, 2H), 3.23-3.21 (m, 2H), 2.79-2.72 (m, 2H), 1.79-1.76 (m, 2H), 1.73-1.66 (m, 1H), 1.58 (td, J = 6.3, 6.5 Hz, 2H), 1.34-1.26 (m, 2H); ^{13}C NMR (125 MHz, DMSO-d_6) δ 178.0, 164.7, 161.2, 156.5, 153.0, 150.7 (dd, J = 12.0, 250.8 Hz), 149.2 (dd, J = 13.3, 244.8 Hz), 138.0, 127.6-127.5 (m), 126.1-126.0 (m), 118.1 (d, J = 18.0 Hz), 117.7 (d, J = 19.1 Hz), 104.4, 98.9, 94.1, 69.7, 43.0, 35.5, 30.0, 28.1; FAB-MS (*m*/*z*) found 418.4 $[M + H]^+$, calcd for $C_{22}H_{21}F_2NO_5$ 417.1.

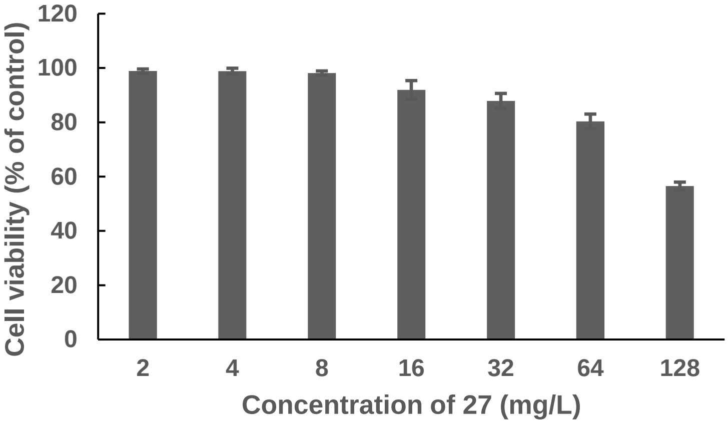

Figure S1. Cell viability of human hepatoma HepG2 cells presented as percentage of control after 24 h exposure to different concentrations of **27**. The data represent the average values of three experiments (± SD).

Table S1. Synergistic anti-biofilm activity of ATM in combination with the multitarget inhibitor **27** against MBL-producing *P. aeruginosa* clinical isolates.

**Isolates, MBLs produced**	Concentrations (mg/L)		Biofilm biomass (OD_{600}) [1]		%-inhibition of biofilm formation [2]
	ATM	**27**	ATM (alone)	ATM/**27**	
**PA-007, *bla*$_{\mathbf{NDM}}$**	1/2 × MIC (8)	32	1.03	0.19	82%
**PA-011, *bla*$_{\mathbf{NDM}}$**	1/2 × MIC (8)	32	0.51	0.17	67%
**PA-012, *bla*$_{\mathbf{NDM}}$**	1/2 × MIC (8)	32	0.23	0.17	24%
**PA-030, *bla*$_{\mathbf{IMP}}$**	1/2 × MIC (32)	32	1.84	0.24	87%

[1] Biofilm biomass was quantified by crystal violet staining at OD_{600}.[2] %-inhibition of biofilm formation = [1 – OD_{600} (ATM/**27**) / OD_{600} (ATM)] × 100
